# *Coptis chinensis* and Myrobalan (*Terminalia chebula*) Can Synergistically Inhibit Inflammatory Response In Vitro and In Vivo

**DOI:** 10.1155/2014/510157

**Published:** 2014-12-18

**Authors:** Enhui Cui, Xiaoyan Zhi, Ying Chen, Yuanyuan Gao, Yunpeng Fan, Weimin Zhang, Wuren Ma, Weifeng Hou, Chao Guo, Xiaoping Song

**Affiliations:** College of Veterinary Medicine, Northwest A&F University, Yangling, Shaanxi 712100, China

## Abstract

*Objectives*. To investigate the anti-inflammatory effect of *Coptis chinensis* plus myrobalan (CM) in vitro and in vivo. *Methods*. The inflammation in mouse peritoneal macrophages was induced by lipopolysaccharide (LPS). Animal models were established by using ear swelling and paw edema of mouse induced by xylene and formaldehyde, respectively. In vitro, cytotoxicity, the phagocytosis of macrophages, the levels of nitric oxide (NO), induced nitric oxide synthase (iNOS), tumor necrosis factor-*α* (TNF-*α*), and interleukin-6 (IL-6) in cell supernatant were detected. In vivo, swelling rate and edema inhibitory rate of ear and paw were observed using CM-treated mice. *Results*. At 150–18.75 *μ*g*·*mL^−1^, CM had no cytotoxicity and could significantly promote the growth and the phagocytosis of macrophages and inhibit the overproduction of NO, iNOS, TNF-*α*, and IL-6 in macrophages induced by LPS. In vivo, pretreatment with CM, the ear swelling, and paw edema of mice could be significantly inhibited in a dose-dependent manner, and the antiedema effect of CM at high dose was better than dexamethasone. *Conclusion*. Our results demonstrated that *Coptis chinensis* and myrobalan possessed synergistically anti-inflammatory activities in vitro and in vivo, which indicated that CM had therapeutic potential for the prevention and treatment of inflammation-mediated diseases.

## 1. Introduction

Inflammation is a physiological process of body that initiates the response to tissue damage or pathogen infection and is always accompanied by the symptoms of redness, swelling, heat, and hyperalgesia [1, 2]. Inflammation includes the participation of various cell types expressing and reacting to a variety of mediators along a precise sequence of events [3]. Inflammation response plays an important role in host survival. Usually, it is activated by inflammatory mediators, such as chemokines and cytokines characterized by recruiting leukocytes to the damage parts [4]. However, the excessive inflammatory mediators participate in many diseases, such as bowel diseases, arthritis, allergic rhinitis, atopic dermatitis, and diverse neurodegenerative diseases [5, 6]. Besides, allergic mediators including histamine, cytokines, and arachidonic acid derivatives could provoke acute and chronic inflammatory responses [7]. Therefore, regulating the production of inflammatory mediator in tissues is very important to the cure of inflammation.

Macrophages are the first defense line of the immune system against pathogen. Increasing evidences indicate that activated macrophages play an important role in inflammatory response through producing various inflammatory factors, such as tumour necrosis factor-*α* (TNF-*α*), interleukin-6 (IL-6), and nitric oxide (NO) [8, 9], which play an important part in the invasion and spread of pathogens in the organism [10]. However, macrophages possess double performance. If macrophages have been in activated state, it could release excessive inflammatory factors, which will injure tissues and cause inflammatory response [11]. Therefore, it is very important to search some drugs for maintaining the balance of immune system though regulating the activity of macrophages and eliminating the inflammatory reaction. Modern pharmacology experiments suggest that inhibiting the inflammatory factors released by macrophages has become the main objective for studying anti-inflammatory drugs [12].

At present, natural product from plant is a promising source for treating the inflammation and allergic reactions [13, 14]. Based on this context, plant-related compounds or extracts can be used for the treatment of inflammatory diseases and development of new anti-inflammatory drugs.* Coptis chinensis*, a member of Ranunculaceae family, derives from the dried rhizome of* Coptis chinensis *Franch.,* Coptis deltoidea *C. Y. Cheng et Hsiao, or* Coptis teeta *Wall.It is a widely used herb in traditional Chinese medicine and attracts much attention because of its multiple pharmacological effects, such as antibacterial, antiviral, anticancer, and antioxidative effects [15]. Berberine is the major active constituent extracted from* Coptis chinensis* [16]. Myrobalan (family Combretaceae) derives from the dried fruits of* Terminalia chebula* Retz. It is recorded in Illustrated Materia Medica; its use could be dated back to more than 1000 years ago. It is the most commonly used in Tibetan Medicine and is called the king of the Tibetan Medicine. Now, myrobalan has been extensively used in Southeast Asia [17]. Tannin, as main active ingredient of myrobalan, has been shown to possess various beneficial pharmacological activities, such as antioxidation, antibacterial, and antivirus activities [18].

After infecting bacteria, the organism often shows inflammatory response. Previous experiments have proved that the prescription,* Coptis chinensis* plus myrobalan (CM), had stronger antibacterial effect in comparison with the single ones [19]. But we did not know whether it possessed a better anti-inflammatory effect. Therefore, in this study, we investigate the anti-inflammatory activity of CM in vitro and in vivo. The aim is to offer theoretical evidence for exploiting new-type anti-inflammatory drugs on curing the inflammation caused by bacteria.

## 2. Materials and Methods

### 2.1. Preparation of CM

*Coptis chinensis* was provided by Anhui Huafeng Chinese Medicine Technology Co., Ltd. Myrobalan was purchased from Hubei Jusheng Science and Technology Co., Ltd. Plant materials (100 g) were extracted with 800 mL of boiling distilled water for 3 h and filtered through a filter paper twice. All the extracts were combined together and centrifuged at 5000 ×g for 30 min. The supernatant was filtered through a 0.22 *μ*m membrane, and the filtrate was then concentrated to 100 mL by evaporation in vacuum at 70°C. Finally, the prescription,* Coptis chinensis* plus myrobalan (CM), was obtained. The concentration of CM was that each 1 mL contained 1 g crude starting materials. In vitro, it was diluted into eleven working concentrations (20 mg·mL^−1^ −19.82 *μ*g·mL^−1^) in twofold serial dilution with RPMI-1640 containing 10% fetal bovine serum, sterilized, and stored at 4°C. In vivo, it was diluted into high (800 mg·mL^−1^), medium (400 mg·mL^−1^), and low (200 mg·mL^−1^) concentrations with deionized water, sterilized, and stored at 4°C. The endotoxin amount was up to the standard of Chinese Veterinary Pharmacopeia (less than 0.5 EU·mL^−1^).

### 2.2. Chemicals and Reagents

Dexamethasone sodium phosphate injection (number 20130722) was purchased from Sichuan Taixin Animal Pharmaceutical Co., Ltd. RPMI-1640 (GIBCO) with the supplement of 100 IU·mL^−1^ benzylpenicillin, 100 IU·mL^−1^ streptomycin, and 10% fetal bovine serum (Hyclone, USA) was used for washing and resuspending cells, diluting mitogen, and culturing the cells. The 3-(4,5-dimethylthiazol-2-yl)-2,5-diphenyltetrazolium bromide (MTT, American Co.) was dissolved into 5 mg·mL^−1^ with calcium and magnesium-free phosphate-buffered saline (PBS, pH 7.2). Lipopolysaccharide (LPS, Sigma, number L2880) was dissolved into 2 *μ*g·mL^−1^ with RPMI-1640. These reagents were filtered through a 0.22 *μ*m millipore membrane filter. Dimethyl sulfoxide (DMSO) was produced by Kemiou Institute of Chemical Engineering in Tianjin, China. Xylene, formaldehyde, and other chemicals used in experiments were of analytical grade.

### 2.3. Analysis of the Berberine in Extract

High performance liquid chromatography (HPLC) analysis was performed using an Agilent Technologies 1200 Series, with a PDA detector and an automatic injector. The column employed was a Zorbax SB-18, 250 × 4.6 mm and 5 *μ*m particle size. The column temperature was set at 30°C. The mobile phase was a mixture of 0.05 moL·L^−1^ KH_2_PO4 in water and acetonitrile (50 : 50, v/v) at a flow rate of 1.0 mL/min. The monitoring wavelength was chosen at 345 nm, and the injection volume was 10 *μ*L. The identification of the compounds was performed by comparison of the retention time and spectrum. The peak area of analyte was used for quantification.

### 2.4. Anti-Inflammatory Activity of CM In Vitro

#### 2.4.1. Peritoneal Macrophages Culture

Macrophages were isolated by peritoneal lavage method with minor modifications according to previous report [20]. In brief, ICR mice (8 weeks old) were injected intraperitoneally with 1 mL of 6% starch-broth medium. Two days later, the peritoneal cavity was washed with 20 mL of PBS, and the macrophages in peritoneal fluid were collected. After centrifugation at 2000 rpm for 10 min, the cells were collected and washed twice with PBS. Then the cells were resuspended in RPMI-1640 and seeded in culture plates (1 × 10^6^ cells·mL^−1^) for 2 h at 37.5°C in a humid atmosphere with 5% CO_2_. Nonadherent cells were removed by light washing twice with RPMI-1640, and the adherent cells were macrophages. Cell viability measured by trypan blue exclusive assay was never below 95%.

#### 2.4.2. Cytotoxicity Analysis

CM was diluted with maintenance medium into 11 concentrations from 20 mg·mL^−1^ to 19.82 *μ*g·mL^−1^ for the test. Then CM at series of concentrations was added into the plates withsplenocytes, 100 *μ*L/well, and four wells each concentration. After a culture for 44 h, 20 *μ*L MTT was added into each well and incubated at 37.5°C in a humid atmosphere of 5% CO_2_ for 4 h, the supernatant was removed, and 100 *μ*L of DMSO was added. The plates were shaken for 5 min to dissolve the crystals completely. The absorbance at 490 nm (*A*_490_ value) of each well was measured by microliter enzyme-linked immunosorbent assay reader. Cell viability was calculated by the following equation: cell viability (%) = Int_*d*_/Int_control_  × 100, where Int_d_: the absorbance in drug group and Int_control_: the absorbance in cell control group [21].

#### 2.4.3. Phagocytic Assay by Neutral Red Method

The phagocytosis of macrophages was measured by neutral red uptake [22]. After being cultured with CM or LPS for 48 h, 100 *μ*L of neutral red solution (0.075%, w/w) was added and incubated for 4 h. After discarding supernatant, the cells were washed with PBS twice to remove the neutral red which was not phagocytized by macrophages. Then DMSO (100 *μ*L/well) was added to lyse macrophages. The absorbance at 490 nm was assayed by a microplate reader.

#### 2.4.4. Measurement of NO

The NO was measured according to the previous description [23]. Cells were pretreated with 2 *μ*g·mL^−1^ of LPS for 2 h and then were cultured with CM at four concentrations (300–37.5 *μ*g·mL^−1^) for 48 h. Finally, 100 *μ*L/well of culture medium was incubated with equal volume of Griess reagent (1% sulfanilamide, 0.1% naphthyl ethylenediamine dihydrochloride, and 2.5% phosphoric acid) for 10 min. NO was determined at 550 nm using ultraviolet spectrophotometer.

#### 2.4.5. Determination of iNOS, TNF-*α*, and IL-6 Levels

The peritoneal macrophages were inoculated into 96-well culture plates. Cells were pretreated with 2 *μ*g·mL^−1^ of LPS for 2 h. After being treated with drugs for 44 h, the plates were centrifuged at 1000 ×g for 10 min, and the supernatant was collected for determining the contents of induced nitric oxide synthase (iNOS), TNF-*α*, and IL-6 by ELISA kit (Biosamite Biotechnology Co. Ltd., Shanghai, China), respectively, according to the manufacturer's instructions.

### 2.5. Anti-Inflammatory Activity of CM In Vivo

#### 2.5.1. Animals

ICR mice (8 weeks old) weighing 20–25 g were purchased from the Fourth Military Medical University Laboratory Animal Center Co. Ltd. (Shanxi, China). They were housed in an environmentally controlled animal facility maintained at 22 ± 2°C with a 12/12 h light/dark cycle. Feed and water were supplied ad libitum. All procedures related to the animals and their care conformed to the internationally accepted principles as found in the Guidelines for Keeping Experimental Animals issued by the Government of China.

#### 2.5.2. Xylene Induced Ear Swelling in Mice

Forty ICR mice (8 weeks old) were randomly divided into four groups. The mice in three experimental groups were treated, respectively, with 1.0 mL of CM at high (800 mg·mL^−1^), medium (400 mg·mL^−1^), and low (200 mg·mL^−1^) dose by gavage, in dexamethasone and blank control (BC) group, 1.0 mL of dexamethasone (0.6 g·kg^−1^), and physiological saline, once a day for 3 successive days. 30 min after the last drug administration, a total volume of 0.05 mL xylene was applied to the anterior and posterior surfaces of the right ear. The left ear was used as control. 1 h later, all mice were euthanized by cervical dislocation and both ears were removed. Circular sections with a diameter of 9 mm were taken using a cork borer and weighed. Edema (Δ_*W*_) is calculated as follows: Δ_*W*_ = *W*_*R*_ − *W*_*L*_, where *W*_*R*_ is the right ear sample weight (mg) and *W*_*L*_ is left ear weight (mg) of the same mouse. Swelling rate and edema inhibitory rate were calculated according to the following formula [24]: swelling rate (%) = Δ_*W*_/*W*_*L*_; inhibitory rate (%) = (*W*_blank control_ − *W*_treated_)/*W*_blank control_ × 100%.

#### 2.5.3. Formaldehyde Induced Paw Edema in Mice

Forty ICR mice (8 weeks old) were randomly divided into four groups. The mice in three experimental groups were treated, respectively, with 1.0 mL of CM at high (800 mg·mL^−1^), medium (400 mg·mL^−1^), and low (200 mg·mL^−1^) dose by gavage, in dexamethasone and blank control (BC) group, 1.0 mL of dexamethasone (0.6 g·kg^−1^), and physiological saline, once a day for 3 successive days. 30 min after the last drug administration, 0.05 mL of formaldehyde was injected into the subplantar area of the right hind paw. The thickness (mm) of the paw was measured before the injection of formaldehyde and then at 3 h after formaldehyde injection. Edema (Δ_*T*_) is calculated as follows: Δ_*T*_ = *T*_*t*_ − *T*_*o*_, where *T*_*t*_ is the right hind paw thickness (mm) after formaldehyde injection and *T*_*o*_ is the right hind paw thickness (mm) prior to subplantar injection. Swelling rate and edema inhibitory rate were calculated according to the following formula: swelling rate (%) = Δ_*T*_/*T*_*o*_; inhibitory rate (%) = (Δ_*T* blank control_ − Δ_*T* treated_)/Δ_*T* blank control_ × 100% [25] (see Figure 9).

### 2.6. Statistical Analysis

Data are expressed as the mean ± S.D. Duncan's multiple range test was used to determine the differences among groups with the software SPSS 19.0. *χ* ^2^-test was used to analyze the difference of the inhibitive rate of edema. Significant differences were considered as *P* < 0.05.

## 3. Results

### 3.1. Characterization of Extracts from CM by HPLC

**Figure 1:**
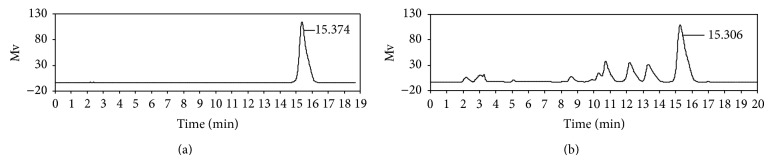
Chromatogram of berberine obtained at 345 nm. (a) Berberine standard; (b) the extract of CM.

Figure 1 shows the base peak chromatogram extract from CM by HPLC. The results showed that berberine standard (20 *μ*g·mL^−1^) only has one peak and the retention time was 15.374 min (Figure 1(a)). At the same location, the extract of CM also had a peak, and the peak possessed a good separating degree (Figure 1(b)). This peak area represented the content of berberine in CM. After being calculated, the content of berberine in CM was 35.8 mg·mL^−1^.

### 3.2. Cytotoxicity

The cell viabilities of every concentration are shown in Figure 1. The cell viabilities of CM at 4800–600 *μ*g·mL^−1^ were significantly smaller than that in cell control (CC) group (*P* < 0.05), which indicated that CM had cytotoxicity and could inhibit the growth of macrophages at these concentrations. The cell viabilities at 150, 4.7, 2.35, and 1.175 *μ*g·mL^−1^ were all slightly larger than that in CC group (*P* > 0.05), which indicated that CM had no cytotoxicity and could not promote cell growth at these concentrations, so 150 *μ*g·mL^−1^ could be considered as maximal safe concentration of CM. The cell viabilities of CM at 150–9.375 *μ*g·mL^−1^ were significantly larger than that in CC group (*P* < 0.5), which indicated that CM could promote the growth of macrophages at these concentrations. In order to investigate the efficacy of CM, therefore, the concentrations of CM were supposed to be 150, 75, 37.5, and 18.75 *μ*g·mL^−1^ for determining its anti-inflammatory activity on macrophages in vitro.

### 3.3. Effect of CM on Phagocytosis of Neutral Red

**Figure 2:**
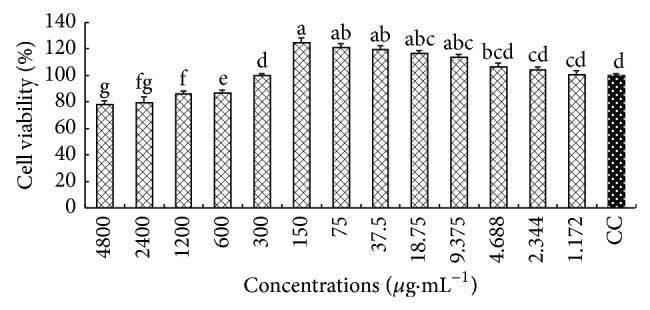
The cell viability of macrophages incubated with CM. ^a-g^Bars without the same superscripts differ significantly (*P* < 0.05).

The effect of CM on phagocytosis of neutral red is illustrated in Figure 2. The* A*_490_ values in LPS and CM groups at 150–18.75 *μ*g·mL^−1^ were significantly higher than CC group (*P* < 0.05). At four concentrations, the* A*_490_ values in CM group were all higher than that in LPS group, and the differences were significant at 312.5 and 156.25 *μ*g·mL^−1^ (*P* < 0.05).

### 3.4. Effect of CM on the Production of NO

**Figure 3:**
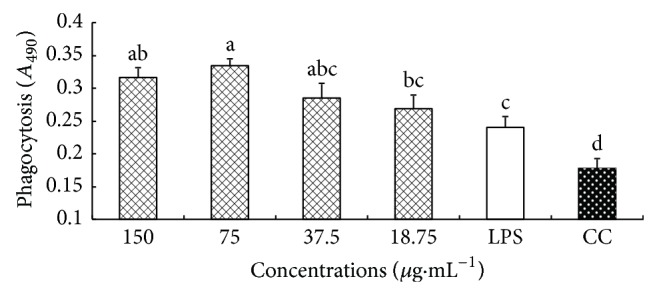
The effect of CM on phagocytosis of macrophages. ^a-d^Bars without the same superscripts differ significantly (*P* < 0.05).

The effect of CM on the production of NO from macrophages is shown in Figure 3. The level of NO in LPS group was significantly higher than that in CC group (*P* < 0.05), which indicated that LPS could stimulate macrophages to produce NO. At 150–18.75 *μ*g·mL^−1^, the levels of NO in CM group were all significantly lower than that in LPS group (*P* < 0.05).

### 3.5. Effect of CM on the Production of iNOS

**Figure 4:**
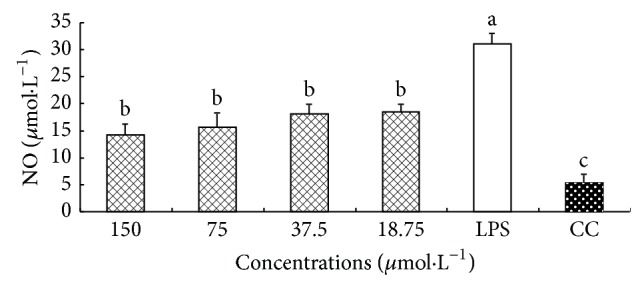
Effect of CM on the production of NO. ^a-c^Bars without the same superscripts differ significantly (*P* < 0.05).

The effect of CM on the production of iNOS from macrophages is shown in Figure 4. The level of iNOS in LPS group was significantly higher than that in CC group (*P* < 0.05). At 150–18.75 *μ*g·mL^−1^, the levels of iNOS in CM group were all significantly lower than that in LPS group (*P* < 0.05).

### 3.6. Effect of CM on TNF-*α* Secretion

**Figure 5:**
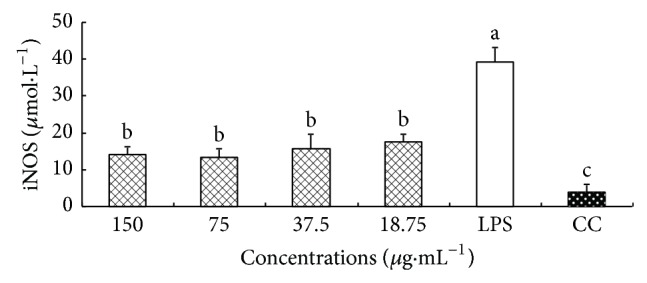
Effect of CM on the production of iNOS. ^a-c^Bars without the same superscripts differ significantly (*P* < 0.05).

The effect of CM on the secretion of TNF-*α* from macrophages is shown in Figure 5. The level of TNF-*α* in LPS group was significantly higher than that in CC group (*P* < 0.05), which indicated that LPS could stimulate macrophages to secrete inflammatory factor (TNF-*α*). At 150–18.75 *μ*g·mL^−1^, the levels of TNF-*α* in CM group were all significantly lower than that in LPS group (*P* < 0.05).

### 3.7. Effect of CM on IL-6 Secretion

**Figure 6:**
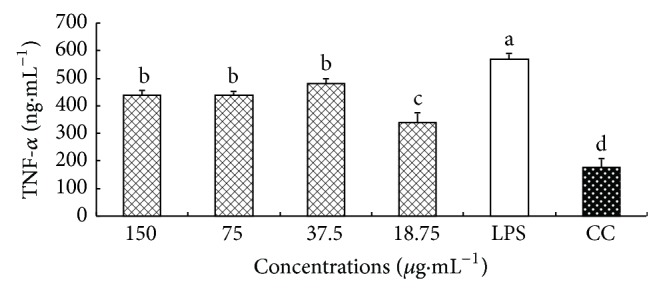
Effect of CM on the TNF-*α* secretion. ^a-d^Bars without the same superscripts differ significantly (*P* < 0.05).

The effect of CM on the secretion of IL-6 from macrophages is shown in Figure 6. The level of IL-6 in LPS group was significantly higher than that in CC group (*P* < 0.05), which indicated that LPS could stimulate macrophages to secrete inflammatory factor (TNF-*α*). At 150–18.75 *μ*g·mL^−1^, the levels of IL-6 in CM group were all significantly lower than that in LPS group (*P* < 0.05).

### 3.8. Effect of CM on Xylene Induced Ear Swelling in Mice

**Figure 7:**
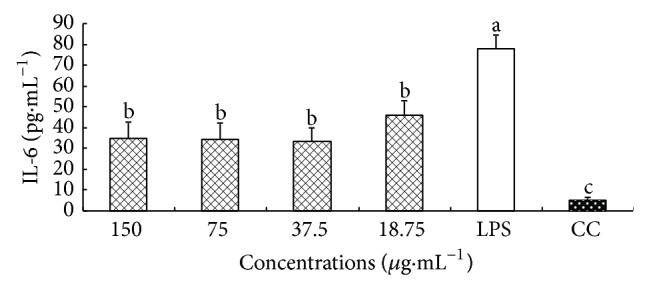
Effect of CM on the IL-6 secretion. ^a-c^Bars without the same superscripts differ significantly (*P* < 0.05).

Figure 7 shows the swelling rate and inhibitory rate of CM in xylene induced ear swelling in mice. The results demonstrated that the swelling rates of ear in CM_H_, CM_M_, CM_L_, and dexamethasone groups were significantly lower than that in BC group (*P* < 0.05). The swelling rate in CM_H_ group was the lowest and significantly lower than other groups (*P* < 0.05). The inhibitory rates of ear in CM_H_, CM_M_, CM_L_, and dexamethasone groups were significantly higher than that in BC group (*P* < 0.05). The inhibitory rate in CM_H_ group was the highest and significantly higher than those in CM_H_ and CM_L_ groups (*P* < 0.05).

### 3.9. Effect of CM on Formaldehyde Induced Paw Edema in Mice

**Figure 8:**
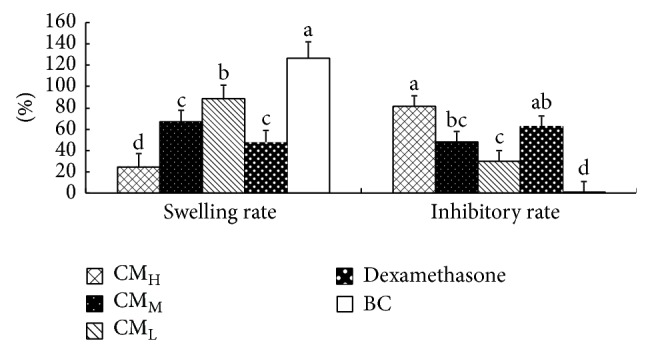
Effect of CM on xylene induced ear swelling in mice. ^a-b^Bars without the same superscripts differ significantly (*P* < 0.05). H: high dose; M: medium dose; L: low dose; BC: blank control.

**Figure 9:**
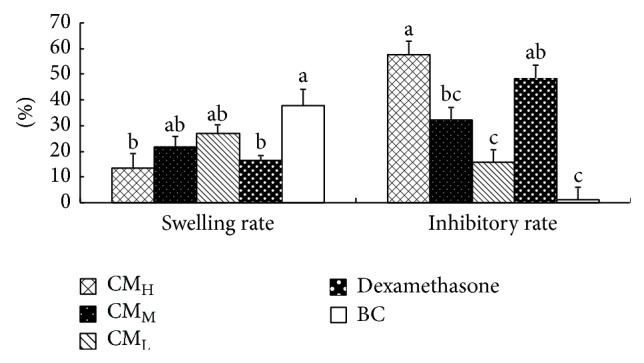
Effect of CM on formaldehyde induced paw edema in mice. ^a-b^Bars without the same superscripts differ significantly (*P* < 0.05). H: high dose; M: medium dose; L: low dose; BC: blank control.

Figure 8 shows the swelling rate and inhibitory rate of CM in formaldehyde induced paw edema in mice. The results demonstrated that the swelling rates of paw in CM_H_, CM_M_, CM_L_, and dexamethasone groups were all lower than that in BC group; CM_H_ group was the lowest and significantly lower than BC group (*P* < 0.05). The inhibitory rates of paw in CM_H_, CM_M_, CM_L_, and dexamethasone groups were all higher than that in BC group; CM_H_ group was the highest and significantly higher than those in CM_M_, CM_L_, and BC groups (*P* < 0.05). There was no significant difference between CM_H_ and dexamethasone groups (*P* > 0.05).

## 4. Discussion

As an important member of natural immune system, macrophage possesses a variety of physiological functions such as antigen presentation, phagocytosis, and stimulating the secretion of inflammatory mediators, and it plays an important role in regulating inflammatory response [26]. LPS possesses a high activity and is a widespread used infectious agent [27]. At present, LPS is considered as the strongest irritant for activating macrophages. LPS can specifically combine with the receptor proteins on the surface of macrophage when it enters the macrophages, then activating the related genes of cytokine and enzyme, finally inducing and synthetizing plenty of inflammatory cytokines [28]. Therefore, macrophage stimulated by LPS has been used as the effective cellular model to study a new drug on anti-inflammatory action and mechanism in vitro [29]. In the present experiment, the peritoneal macrophages of mouse stimulated with LPS were used to investigate the anti-inflammatory activity of CM. The experimental results showed that the contents of NO, iNOS, TNF-*α*, and IL-6 in LPS group were significantly higher than those in blank control group, which indicated that the inflammatory model was established successfully. So this model could be used to evaluate the anti-inflammatory effect of CM.

Phagocytosis is the indispensable step of the immunological defense system. The enhancement of phagocytic function is expected to be applicable for treating microbial infections and cancer, because phagocytes play roles as regulatory and effector cells by phagocytizing pathogenic microorganism and invasive cells (including cancer cells) in immune system [30]. In this study, we detected the phagocytosis activity of CM on macrophages. The experimental results showed that CM could significantly increase the phagocytosis of macrophages, and the effect was significantly higher than LPS. These results suggested that CM could result in the initiation of immune reaction against foreign materials such as pathogens and tumors. Many researches also proved that some traditional Chinese medicines could improve the phagocytosis activity of peritoneal macrophages [31, 32].

Nitric oxide (NO), as an important second messenger molecule in mammalian, participates in various physiological and pathological processes. Appropriate levels of NO in body are essential in maintaining many normal physiological functions, but excessive NO possesses cytotoxicity and could result in sepsis, inflammation, and carcinogenesis [33]. iNOS is the most important enzyme to catalyse NO after the activation of macrophages [34]. Therefore, it is conducive to estimate the effect of drugs on inhibiting the early inflammatory response through studying the production of NO and iNOS in macrophages. The experimental results showed that CM could significantly inhibit the release of NO and iNOS in macrophages stimulated by LPS; it suggested that CM had a better anti-inflammation effect. The reason may be that CM inhibited the level of NO production through suppressing the activity or overexpression of iNOS. Many researches proved that some herb medicines possessed anti-inflammatory activity and also could inhibit the release of NO and iNOS in inflammatory response [35, 36].

TNF-*α* and IL-6 are the important cytokines in the early pathogenesis of inflammation. In inflammatory response, TNF-*α* could induce nonspecific immune responses by activating macrophages and stimulating the secretions of other inflammatory cytokines [37]. It is released during the early phase of acute and chronic inflammatory diseases, such as septic shock, rheumatoid arthritis, and other allergic diseases. IL-6, mainly produced by T cells and macrophages, is considered as one kind of markers of the inflammation [36]. At the beginning of the inflammation, IL-6 is expressed in abundance; it is involved in the further development of the inflammatory response and induces the expression of other inflammatory factors. Therefore, IL-6 plays an important role in inflammatory response [9]. In our experiment, CM could significantly reduce the production of TNF-*α* and IL-6 in macrophages induced by LPS. It indicated that CM had a better anti-inflammatory effect. The biosynthesis of TNF-*α* and IL-6 is regulated and controlled by complex and multiple mechanisms, which mainly includes gene transcription, mRNA turnover, and translation of intracellular signaling of proteins [38]. Therefore, CM with the inhibitory the secretion of TNF-*α* and IL-6 might be possible to target these aspects.

Edema is a kind of basic pathological changes at the inflammatory site, and it also is the character of acute inflammation. The effect of anti-inflammatory drug greatly depends on its inhibition degree on edema when it was used in acute inflammation. In this experiment, the animal models including ear swelling and paw edema induced by xylene and formaldehyde, respectively, are used to investigate the anti-inflammatory activity of CM. The model of ear swelling induced by xylene is a classical animal model for assessing the anti-inflammatory activity of drugs, because xylene could stimulate the production of inflammatory mediators such as bradykinin, prostaglandins, and serotonin, which induce ear swelling by promoting vasodilation and increasing vascular permeability [39]. The results showed that CM could dose-dependently inhibit the formation of edema induced by xylene, and the effect was better than dexamethasone at high dose. The reason may be that CM interfered with the secretion of the above mediators. As an acute inflammatory model, paw edema induced by formaldehyde is also widely used to evaluate the anti-inflammatory activity of drugs. This process includes three stages [40]. The final stage (2.5–6 h) is the most important process in the inflammatory response, because it is associated with leukocyte migration into the inflamed site [41]. So we determined the paw edema at 3 h after injecting formaldehyde. The result indicated that CM played a crucial role in protecting against acute inflammation induced by formaldehyde, and the effect was better than dexamethasone at high dose.

## 5. Conclusions

The results demonstrated that CM had potent anti-inflammatory activity in vitro and in vivo, including promoting the phagocytic function of macrophages, inducing the release of NO, iNOS, TNF-*α*, and IL-6 by macrophages, and inhibiting the edema of ear and paw in mice. Nevertheless, the mechanism of the effect on macrophages by CM is still unknown; further studies and experiments on the mechanism of CM in vivo are in progress.

## Abbreviations

CM: * Coptis chinensis* plus myrobalan
TNF-*α*: Tumour necrosis factor-*α*
IL-6: Interleukin-6
NO: Nitric oxide
LPS: Lipopolysaccharide
MTT: 3-(4,5-Dimethylthiazol-2-yl)-2,5-diphenyltetrazolium bromide
PBS: Phosphate buffered saline
DMSO: Dimethyl sulfoxide
iNOS: Induced nitric oxide synthase
BC: Blank control
CC: Cell control.
